# Attenuation measurements show that the presence of a TachoSil surgical patch will not compromise target irradiation in intra-operative electron radiation therapy or high-dose-rate brachytherapy

**DOI:** 10.1186/s13014-014-0316-1

**Published:** 2015-01-09

**Authors:** Sandra Sarmento, Filipa Costa, Alexandre Pereira, Joana Lencart, Anabela Dias, Luís Cunha, Olga Sousa, José Pedro Silva, Lúcio Santos

**Affiliations:** Medical Physics Department, Portuguese Institute of Oncology, Porto, Portugal; Medical Physics, Radiobiology and Radiation Protection Group, Research Centre, Portuguese Institute of Oncology, Porto, Portugal; Radiation Oncology Department, Portuguese Institute of Oncology, Porto, Portugal; Surgical Oncology Department, Portuguese Institute of Oncology, Porto, Portugal; Experimental Pathology and Therapeutics Group, Research Centre, Portuguese Institute of Oncology, Porto, Portugal

**Keywords:** Intra-operative electron radiation therapy, TachoSil, Haemostatic patch, Rectal cancer

## Abstract

**Background:**

Surgery of locally advanced and/or recurrent rectal cancer can be complemented with intra-operative electron radiation therapy (IOERT) to deliver a single dose of radiation directly to the unresectable margins, while sparing nearby sensitive organs/structures. Haemorrhages may occur and can affect the dose distribution, leading to an incorrect target irradiation. The TachoSil (TS) surgical patch, when activated, creates a fibrin clot at the surgical site to achieve haemostasis. The aim of this work was to determine the effect of TS on the dose distribution, and ascertain whether it could be used in combination with IOERT. This characterization was extended to include high dose rate (HDR) intraoperative brachytherapy, which is sometimes used at other institutions instead of IOERT.

**Methods:**

CT images of the TS patch were acquired for initial characterization. Dosimetric measurements were performed in a water tank phantom, using a conventional LINAC with a hard-docking system of cylindrical applicators. Percentage Depth Dose (PDD) curves were obtained, and measurements made at the depth of dose maximum for the three clinically used electron energies (6, 9 and 12MeV), first without any attenuator and then with the activated patch of TS completely covering the tip of the IOERT applicator. For HDR brachytherapy, a measurement setup was improvised using a solid water phantom and a Farmer ionization chamber.

**Results:**

Our measurements show that the attenuation of a TachoSil patch is negligible, both for high energy electron beams (6 to 12MeV), and for a HDR ^192^Ir brachytherapy source. Our results cannot be extrapolated to lower beam energies such as 50 kVp X-rays, which are sometimes used for breast IORT.

**Conclusion:**

The TachoSil surgical patch can be used in IORT procedures using 6MeV electron energies or higher, or HDR ^192^Ir brachytherapy.

## Background

In intra-operative radiation therapy (IORT), ionizing radiation is used during a surgical intervention [1,2]. After removal of the neoplastic mass, the remaining area is irradiated for direct treatment of the resection margins, including positive margins, according to international protocols [1].

Surgery of locally advanced and/or recurrent rectal cancer can be complemented with intra-operative electron radiation therapy (IOERT). Sometimes, high-dose-rate brachytherapy (IO-HDR) with a source of Iridium- 192 (^192^Ir) is used instead of IOERT [3]. Both modalities deliver a single radiation dose directly to the malignant tissues, while sparing nearby sensitive organs/structures [1,2].

Haemorrhages may occur in the pelvic area during a surgery, or a haematic fluid build-up, which may affect the dose distribution during irradiation if no haemostatic measures are applied [4]. At our institution, surgery of locally advanced and/or recurrent rectal cancer is often complemented with IOERT, and fluid build-up is frequently observed. The solution adopted is to use constant suction. However, a residual accumulation of fluid generally remains.

TachoSil (Takeda Pharmaceuticals, Zurich, Switzerland) is an absorbable fibrin sealant patch indicated for haemostasis during surgical procedures [5,6]. To the authors’ knowledge, it has never been used in combination with IORT, and its radiation attenuation properties have never been measured or reported.

Since TachoSil (TS) has already proven useful in situations of pelvic bleeding [7,8], it seems reasonable to suppose that this sealant patch could be beneficial in IORT, as it would help minimize the fluid build-up by reducing the bleeding in or near the irradiation target. Before initiating clinical trials to assess its efficacy, safety and clinical relevance, it is necessary first to measure the attenuation properties of the TS patch, in order to determine whether its presence will affect the dose distribution. This is particularly important in IOERT, where dose calculations are performed manually, after visual estimation of the target area. More sophisticated forms of treatment planning would require computed tomography (CT) images of the patient, which are difficult to obtain during a surgical procedure. Therefore, any additional attenuation must be known in advance so it can be taken in consideration during calculations.

The aim of this work is to characterize the effect of a TachoSil (TS) patch on the dose distribution underneath it, through detailed dosimetric measurements.

## Methods

The TS patch is a white collagen sponge coated on one side with a yellow layer of active components from human blood, fibrinogen and thrombin. When the sponge comes into contact with fluids (such as blood, lymph or saline solution) the fibrinogen and the thrombin are activated and form a fibrin network. This means the sponge sticks to the tissue surface, the blood coagulates (local haemostasis) and the tissue is sealed [8].

**Figure 1:**
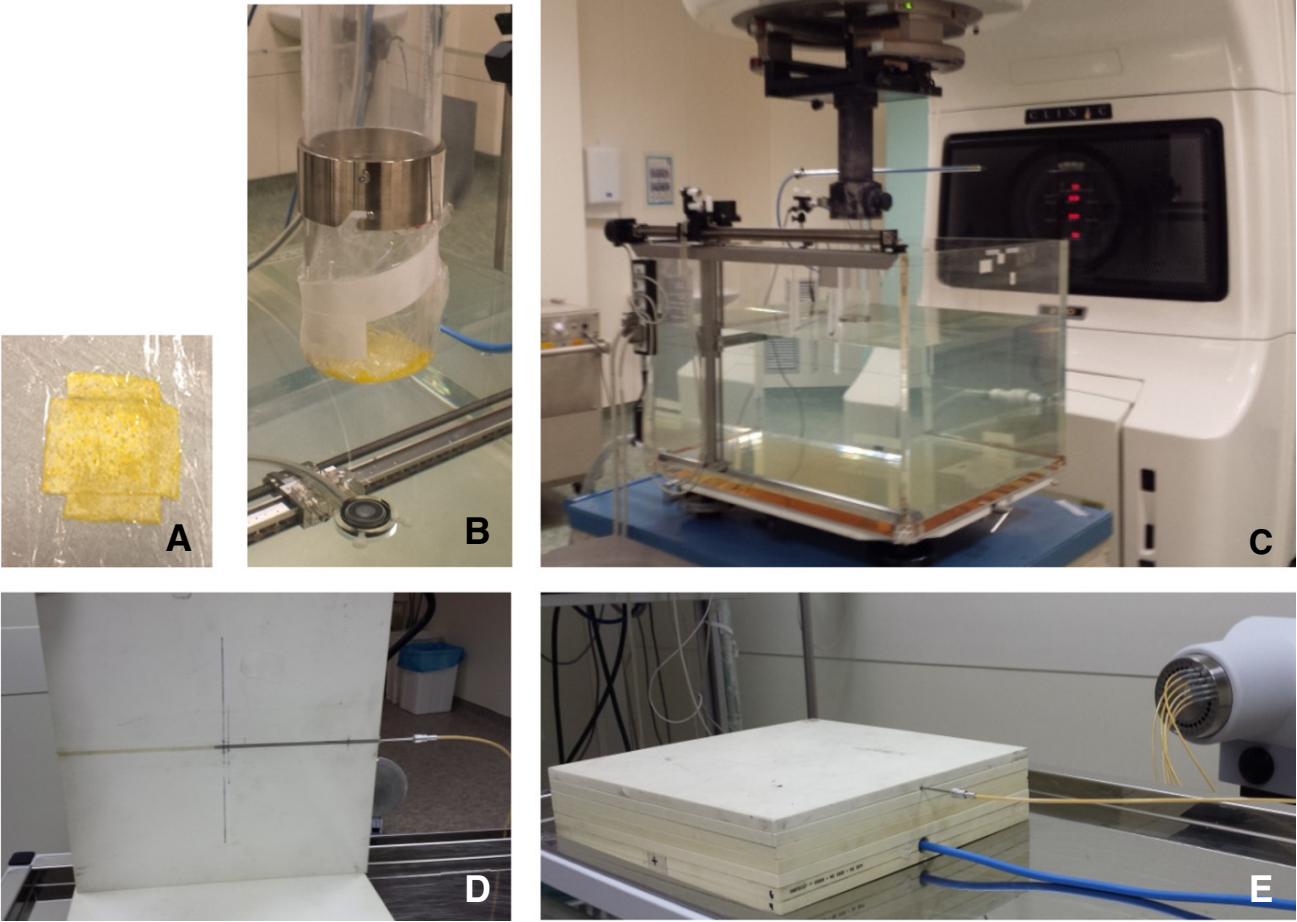
**Experimental setup for dosimetric measurements with TachoSil in IOERT and in HDR** ^**192**^ **Ir brachytherapy. (A)** Activated TachoSil patch wrapped in very thin plastic film. **(B)** Same patch covering the tip of a 7 cm IOERT applicator. **(C)** Water tank used for dosimetric measurements, and the conventional LINAC adapted for IOERT with a hard-docking system of applicators. **(D)** Phantom slab adapted to hold HDR needle **(E)** Set-up used to study the effect of TachoSil in treatments with ^192^Ir.

The inactivated (dry) patch was cut to size, activated with saline solution and wrapped in very thin plastic film, to permit easy manipulation (Figure 1A). Therefore, the patch could be held in place at the end of an IOERT applicator, as shown in Figure 1B, both for dosimetric measurements and for acquisition of computed tomography (CT) images. For initial characterization, CT images of dry and activated TS were obtained using a Toshiba Asteion 4-slice CT scanner in axial mode.

IORT procedures at our institution are always performed with high energy electron beams (IOERT), using a conventional linear accelerator (Varian 2100CD), with a hard-docking system of cylindrical applicators, with diameters ranging from 3 to 15 cm and bevel angles of 0, 15, 30 and 45° (Figure 1C). The available electron energies are 6, 9, 12, 15 and 18MeV, but only 6, 9 and 12MeV are used for IOERT.

To determine the effect of TS on the IOERT dose distribution, dosimetric measurements were performed in a water tank phantom (MP3, PTW-Freiburg, Germany). The applicator of 7 cm diameter, bevel angle of 0° was used for the measurements, with source to surface distance (SSD) of 134 cm. This is the reference SSD for this IOERT applicator system (usable SSDs range between 124 and 144 cm). Measurements were performed with an activated patch completely covering the IOERT applicator opening (Figure 1B) and without it. During measurements, either the tip of the applicator, or the TachoSil covering it, were in contact with the water surface (air gap = 0). Percentage Depth Dose (PDD) curves were obtained for the three clinically used electron energies (6, 9 and 12MeV) with a diode detector (type 60012 E PTW-Freiburg, Germany). A Markus ionization chamber (type N23343 PTW-Freiburg, Germany) was then placed at the depth of dose maximum (Dmax), and readings were obtained for 6, 9 and 12MeV, first without attenuator and then with the activated patch covering the tip of the applicator.

HDR brachytherapy with ^192^Ir is commonly performed at our institution, although not for IORT. For a more complete characterization and to anticipate future possibilities, this modality was also considered in the present study. Dosimetry for HDR brachytherapy is not usually done in water phantom, therefore a measuring setup was improvised using a white polystyrene phantom, also known as solid water phantom (type RW3 PTW-Freiburg, Germany). As shown in Figure 1D, one of the phantom slabs was adapted to hold a brachytherapy applicator (needle), connected by a cable to the afterloader device, which was programmed to position the ^192^Ir source at the centre of the slab, for 60s, for each measurement. The detector used was a Farmer ionization chamber (type 30006 PTW-Freiburg, Germany), placed inside the appropriate phantom slab. Four 1 cm slabs were added between the slab containing the needle and the one containing the Farmer chamber, as shown in Figure 1E. The activated patch was placed at the centre, between the second and third uniform slabs. Spacers less than 1 mm thick were added so that measurements could be performed with and without attenuator, at the same source-to-detector distance.

## Results and discussion

### CT images

CT images provide information about the way a material interacts with ionising radiation. This interaction depends both on the electronic density of the material, and on the type and energy of the incident radiation. Therefore the relationship between CT numbers and electronic density varies slightly between scanners and acquisition protocols [9]. But within each CT image, materials with similar electronic density will have similar CT numbers, which in the context of this work is enough for comparison purposes. Typical CT numbers of relevant materials are −1000 HU for air, 0 HU for water, +92 HU to +137 HU for acrylic, +100 HU to +300 HU for soft tissue and −100 HU to – 50 HU for fat.

**Figure 2:**
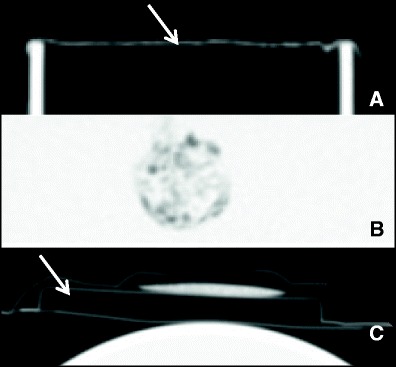
**CT images of non-activated and activated TachoSil. (A)** Activated TachoSil placed on the tip of a 7 cm IOERT acrylic applicator. **(B)** Same patch folded and immersed in water **(C)** Non-activated TachoSil inside its package, placed on top of a cylindrical acrylic phantom filled with water. The desiccant included in the package is also clearly visible. The TachoSil patch is indicated by a white arrow.

CT images of non-activated (dry) and activated (wet) TS were obtained and are presented in Figure 2. CT numbers for the non activated TachoSil patch are between −980 HU and −900 HU (Figure 2C). The non activated patch is originally 3 to 4 mm thick, but we observed that this thickness is reduced to less than 1 millimetre when the patch is activated. It is difficult to measure CT numbers accurately for such a thin strip of material. The CT numbers for the activated patch appear to be −100 HU in Figure 2B and −500 HU in Figure 2A. These results, and the visual analysis of the images (Figure 2), suggest that the electronic density of the patch increases with the activation process, probably due to the absorption of fluid, but remains lower than that of acrylic or water, and consequently lower than that of soft tissue.

### Attenuation of high energy electron beams (IOERT)

**Figure 3:**
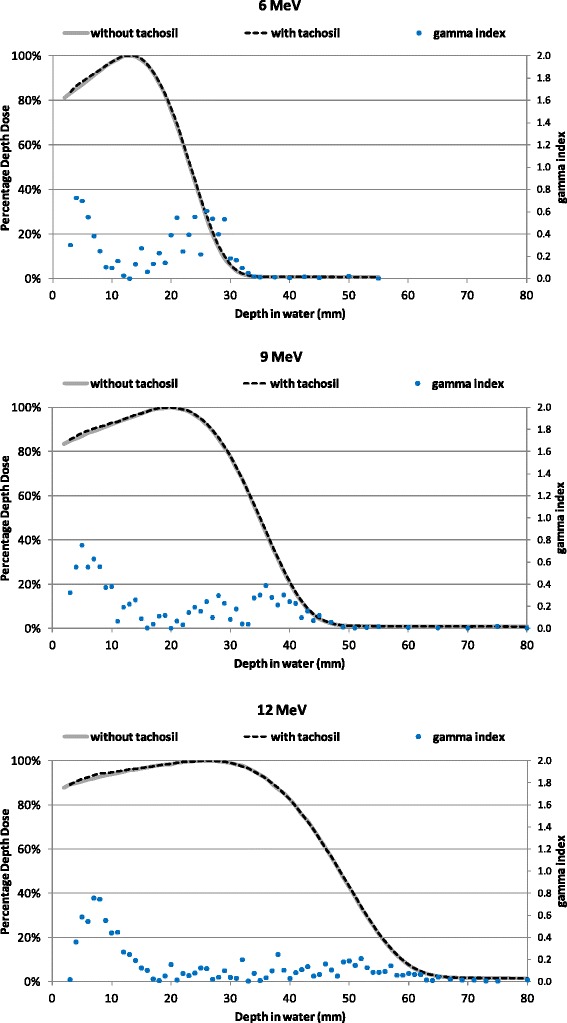
**PDDs with and without TS for 7 cm applicator and 6, 9 and 12MeV electron energies.** Differences between PDDs with and without TS were evaluated by gamma function analysis (2%, 2 mm criteria), and the gamma (γ) index is plotted in the secondary axis.

The Percentage Depth Dose (PDD) curves measured in water phantom without and with the TachoSil patch are shown in Figure 3 for all three electron energies. No significant difference was observed in PDDs obtained with and without this attenuator. This is confirmed by applying a gamma (γ) index analysis to compare both PDD curves [10]. The gamma index distribution is presented in Figure 3, plotted in the same graph as the PDDs. Acceptable limits for the distance and dose differences were set to 2 mm and 2%, respectively. 100% of points pass the gamma test (γ ≤ 1) with these criteria.

The similarity of the PDD curves is consistent with little or no attenuation of the TS patch. This is in good agreement with the results presented in the previous section, which indicate that the activated patch is a very thin (<1 mm) strip of material with electronic density lower than that of tissue.

The results of measurements performed with the Markus ionization chamber at the depth of dose maximum (Dmax) are presented in Table 1, as chamber readings corrected for the influence quantities temperature and pressure. Readings were obtained first without attenuator (M) and then with an activated patch covering the applicator end (M_T_).

**Table 1:** **Markus ionization chamber readings without (M) and with TachoSil (M**_T_ **)**

**Energy (MeV)**	**Dmax (mm)**	**M (nC)**	**M**_T_ **(nC)**	**Dif**_T_
6	13	2.259 ± 0.003	2.258 ± 0.003	−0.1%
9	20	2.714 ± 0.003	2.716 ± 0.003	0.1%
12	27	3.015 ± 0.003	3.018 ± 0.003	0.1%

Markus readings corrected for the influence quantities of temperature and pressure. Indicated uncertainty corresponds to the maximum fluctuation of Markus chamber readings between measurements under the same conditions. All M_T_ vs M differences are within the experimental uncertainty of ± 0.003 nC or ±0.1%.

No significant differences were observed in absolute measurements with and without TachoSil. All measured variations are within the experimental uncertainty of ±0.1%, confirming that the attenuation of the sealant patch is negligible for high energy electron beams of 6 to 12MeV.

The displacement of water caused by the presence of the sealant patch at the tip of the IOERT applicator was minimal. Nevertheless, to confirm that water displacement did not mask the attenuation, the measurements at the depth of dose maximum were repeated in a solid water phantom, for 6 and 12MeV, with similar results. The highest variation observed (−0.2% for 6MeV) was still within experimental error (±0.1%).

According to these results, this surgical patch can be used in combination with intra-operative electron radiation therapy (IOERT), for electron energies of 6MeV or higher, without any significant beam attenuation or alteration of dose distribution.

### Attenuation of ^192^Ir (IO-HDR)

Percentage Depth Dose (PDD) curves were not obtained for this modality, due to the different experimental setup used. The Farmer ionization chamber readings obtained, corrected for the influence quantities temperature and pressure, are presented in Table 2.

**Table 2:** **Farmer ionization chamber readings without (M) and with TachoSil (M**_T_ **)**

**M (nC)**	**M**_T_ **(nC)**	**Dif**_T_
3.486 ± 0.001	3.469 ± 0.001	−0.5%

When a TachoSil patch is placed between the phantom slabs, the resulting attenuation is ~0.5%. This is higher than observed for high energy electron beams, but still lower than the admissible uncertainty for radiotherapy output dose (2%). Even if more than one sealant patch is used, with overlapping, the combined effect will still be negligible. There is no need to account for added attenuation during treatment planning.

### Further considerations

According to the manufacturer, TachoSil is sterilized by gamma irradiation after completion of inner and outer packaging [11]. This sterilization is performed with a source of Cobalt-60 (^60^Co) delivering doses of the order of kGy [12]. Therefore, the effect of radiation on the surgical patch itself was not studied, since it has already been subjected to doses much higher than the 10–20 Gy typically used in IORT treatments. Moreover, patch activation and sealing effect will occur before the IORT irradiation.

Intra-operative electron radiation therapy (IOERT) can be performed with conventional LINACs like ours, or with dedicated mobile units such as NOVAC7, Mobetron and LIAC. Mobile LINACs have higher dose rates [13,14] than conventional LINACs, but operate in a similar range of electron energies. For this reason, mobile LINACs were not considered separately in the context of this work.

At our institution, 9MeV is the most commonly used electron energy for pelvic IOERT, so the attenuation of the sealant patch was determined only for electron energies of 6 to 12MeV. It is important to note that these attenuation results cannot be extrapolated to lower electron energies, and especially not for low energy X-rays, which are sometimes used for breast IORT [15].

A preliminary assessment using a 50 kVp X-ray beam from a conventional radiodiagnostic unit suggests that, at such low beam energies, the attenuation could be as high as 6%. Therefore, TachoSil must not be used in IORT with low energy X-rays without further investigations, which fall outside the scope of the present work.

## Conclusion

The activated TS patch is less than 1 millimetre thick. Even after activation, its electronic density remains lower than that of tissue. Our measurements confirm that the attenuation of the TS patch is negligible for electron energies between 6MeV and 12MeV. Percentage Depth Dose (PDD) curves obtained with and without attenuator are practically coincident, and dose readings at Dmax remained constant within experimental uncertainty. Moreover, the attenuation of the TS patch was found to be ~0.5% for a HDR ^192^Ir brachytherapy source, which is still negligible for the purpose of treatment planning.

Therefore, the TachoSil surgical patch can be used during IOERT and IO-HDR procedures, since it will not alter the dose distribution. Our next step is to ascertain the effectiveness, safety and clinical usefulness of this sealant patch during IOERT of the pelvic region. Hopefully it will help control the build-up of fluid affecting the dose distribution, by minimizing the bleeding near the irradiation target.
