# Erratum: Attenuation measurements show that the presence of a TachoSil surgical patch will not compromise target irradiation in intra-operative electron radiation therapy or high-dose-rate brachytherapy

**DOI:** 10.1186/s13014-015-0565-7

**Published:** 2015-12-15

**Authors:** Sandra Sarmento, Filipa Costa, Alexandre Pereira, Joana Lencart, Anabela Dias, Luís Cunha, Olga Sousa, José Pedro Silva, Lúcio Santos

**Affiliations:** Medical Physics Department, Portuguese Institute of Oncology, Porto, Portugal; Medical Physics, Radiobiology and Radiation Protection Group, Research Centre, Portuguese Institute of Oncology, Porto, Portugal; Radiation Oncology Department, Portuguese Institute of Oncology, Porto, Portugal; Surgical Oncology Department, Portuguese Institute of Oncology, Porto, Portugal; Experimental Pathology and Therapeutics Group, Research Centre, Portuguese Institute of Oncology, Porto, Portugal

After publication of this study [1], the authors noticed that the funding was incorrectly acknowledged. The correct Acknowledgements section can be found below:

“This work was partly funded by Fundação para a Ciência e Tecnologia (FCT), in the framework of the project PTDC/SAU-ENB/117631/2010, which is cofinanced by FEDER, through Programa Operacional Fatores de Competitividade - COMPETE of QREN (reference FCOMP-01-0124-FEDER-021141).”
